# Herpes zoster vaccine (HZV): utilization and coverage 2009 – 2013, Alberta, Canada

**DOI:** 10.1186/1471-2458-14-1098

**Published:** 2014-10-23

**Authors:** Xianfang C Liu, Kimberley A Simmonds, Margaret L Russell, Lawrence W Svenson

**Affiliations:** Department of Community Health Sciences, University of Calgary, 3280 Hospital Drive NW, Calgary, Alberta T2N 4Z6 Canada; Epidemiology and Surveillance Team, Alberta Ministry of Health, 23rd fl Telus Plaza NT, 10025 Jasper Avenue, Edmonton, Alberta T5J 1S6 Canada; School of Public Health, University of Alberta, Edmonton, Alberta T6G 1C9 Canada

## Abstract

**Background:**

Herpes zoster vaccine (HZV) is not publicly funded in the province of Alberta, Canada. We estimated vaccine coverage among those aged 60 years or older for 2013, as well as vaccine utilization rates per hundred thousand population over the period 2009 – 2013. We explored for factors associated with HZV dispensing rates.

**Methods:**

We used administrative data from the Alberta Pharmaceutical Information Network (PIN) database to identify unique persons for whom HZV had been dispensed from community pharmacies over 2009 – 2013. PIN data were also used to estimate the pharmacy/population ratios for rural and urban Alberta over the period. Denominators for rates were estimated using mid-year population estimates from the Alberta Health Care Insurance Plan Registry. Income quintile data were estimated from the 2006 Census of Canada. Crude, age, sex, geographic (rural vs. urban), income-quintile and year specific rates of HZV vaccine dispensing were estimated per 100,000 population. Rates were adjusted for pharmacy/population ratio. Vaccine coverage for persons aged 60 years or older was estimated using counts of all unique persons for whom the vaccine was dispensed over the period in the numerator and a 2013 mid- year population denominator.

**Results:**

HZV dispensing rates rose annually from 2009 – 2013. Vaccine coverage was estimated to be 8.4% among persons aged 60 years or older. Rates of dispensing were highest for persons aged 60–69 years and were higher for females than males and for persons from higher compared to lower income quintiles. Dispensing rates were lower for rural than for urban residents. About 2% of vaccine was dispensed for persons aged less than 50 years.

**Conclusions:**

Rates of HZV dispensing are increasing rapidly in Alberta despite a lack of public funding. A small proportion of the vaccine may be dispensed off-label.

## Background

Monitoring trends in both disease and vaccination are important components of disease control programs [1] for vaccine preventable diseases. Varicella-zoster virus causes two distinct diseases: varicella (chickenpox) and herpes zoster (shingles). Different vaccines have been developed for the prevention of these diseases: varicella vaccines for the prevention of chickenpox, and herpes zoster vaccine (HZV) for the prevention of shingles [2]. Shingles can occur both after a primary infection with wild varicella virus and after vaccination with live attenuated varicella vaccine; although the risk of shingles, at least in healthy vaccinated children, is less than after having chickenpox disease, and the disease milder [3, 4]. To monitor varicella zoster virus, surveillance programs should include surveillance for both diseases and population coverage for both vaccines.

In Alberta, Canada, a publicly funded varicella vaccination program for chickenpox has existed since 2001; a single dose is recommended for children age 12 months or older and coverage has exceeded 80% since 2005 [5]. Current immunization rates in Alberta are estimated to be just under 85% [6]. Other studies have addressed the epidemiology of chickenpox [7, 8] and shingles [5, 8, 9] in the periods prior to and after public funding of chickenpox vaccine. In 2008, a live attenuated HZV was authorized for use in Canada for the prevention of herpes zoster infection in adults aged 60 years and older [10]; and the authorization subsequently expanded to include persons aged 50 years or older in 2011 [11]. The vaccine was licensed in the U.S.A. in 2006 and the United States Advisory Committee on Immunization Practices recommends it only for persons aged 60 years or older [12, 13]. In 2010, the Canadian National Advisory Committee on Immunization (NACI) recommended that the vaccine be used for all persons aged 60 years or older who did not have a contra-indication, and indicated that it may be used in persons aged 50 years and older [10]. The vaccine is administered as a single dose. Although the vaccine is not publicly funded in any Canadian province or territory [14], it is available for private purchase in Alberta through community pharmacies. We examine the epidemiology of population utilization of HZV vaccine (Zostavax™) in Alberta over the period of 2009 – 2013 using population-based dispensing data for community pharmacies. We estimate HZV coverage among persons aged 60 years or older, as well as for persons aged 50 years or older for 2013.

## Methods

We used administrative health data from the Alberta Pharmaceutical Information Network (PIN) database to capture the number of unique persons for whom HZV vaccine was dispensed in Alberta as well as a count of the number of pharmacies with a dispense event for any prescription pharmaceutical in each year for the period 2009 – 2013. The study was approved by the University of Calgary Conjoint Health Research Ethics Board (Protocol 23776). Written informed consent from participants was not required as the data custodians released only anonymous aggregated data to the investigators. In Alberta, hospital pharmacies dispense only to patients while they are hospitalized; prescription pharmaceuticals (including prescriptions that are written at the time of hospital discharge) are otherwise dispensed from community pharmacies. All pharmacists working in community pharmacies are mandated to submit drug dispensing information to the Alberta Ministry of Health for entry into PIN since September 1, 2007 [15]. PIN is estimated to capture information on more than 95% of the drugs and biologicals dispensed by community pharmacies [16]. This system, updated on a daily basis, captures information on dispensing of vaccines and drugs, as well as a unique personal identifier (PHN) for the person for whom the vaccine/drug is dispensed and a unique identifier for the pharmacy from which the drug or vaccine was dispensed. The system captures all medications dispensed by the pharmacy regardless of patient insurance status [17]. The PHN can be linked to the Alberta Health Care Insurance Plan Registry from which the date of birth and address of the individual were obtained. Postal codes of residence that included a zero in the second place of the six-digit postal code were classified as rural [18, 19]. Rural and urban income quintiles for Alberta (aggregated at the level of postal code) were calculated from 2006 Canada census data using methods similar to those of Martens and colleagues [20]. File linkage and data extraction were conducted by employees of Alberta Health (data custodians) for the period 2009–2013, and the data aggregated for privacy protection prior to release to the researchers.

Denominators for rates were estimated using mid-year population estimates from the Alberta Health Care Insurance Plan Registry [21] which has been shown to be a reliable population data source [22]. Data were analyzed using SAS 9.3 (SAS Institute Inc., Cary, NC 2011) and graphs created using SigmaPlot Version 12.5 (Systat Software, San Jose, CA 2013). Annual age-, sex-, geography-, and income quintile-specific rates were estimated. A population level HZV coverage rate was estimated by taking all individuals filling a prescription for vaccine during the study period and linking them with the 2013 mid-year population file from the Alberta Health Care Insurance Plan Registry. This approach allowed for the removal of individuals who died or moved out of province.

The crude relative risk of HZV vaccine dispensing rates was calculated using modified Poisson regression with robust error variances [23]. The reference group used in the analysis was the 50–54 year old age group. Negative binomial regression was used to explore for age group (5-year age-group), sex, geography (rural vs. urban), income quintile, pharmacy/population ratio and year effects and their interactions for persons aged 50 years or older. A forward stepwise approach was used to construct a model with the best fit where all terms were significant predictors of the outcome at alpha = 0.05. To a model that contained main effects for each of age-group, sex, geography, year, income quintile and pharmacy/population ratio we added the two-way interaction terms sequentially, at each step examining the likelihood ratio statistic at alpha = 0.05.

## Results

**Figure 1:**
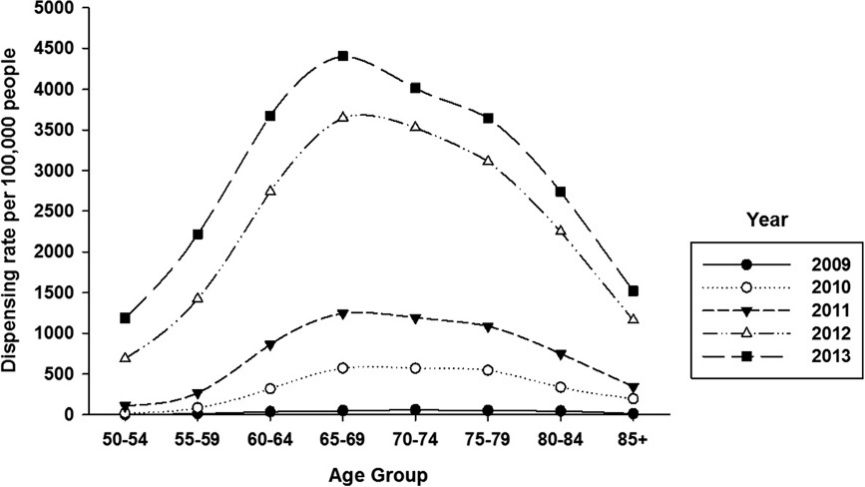
**HZV dispensing rates by age-group and year, persons aged 50 years or older, Alberta 2009–2013.**

**Figure 2:**
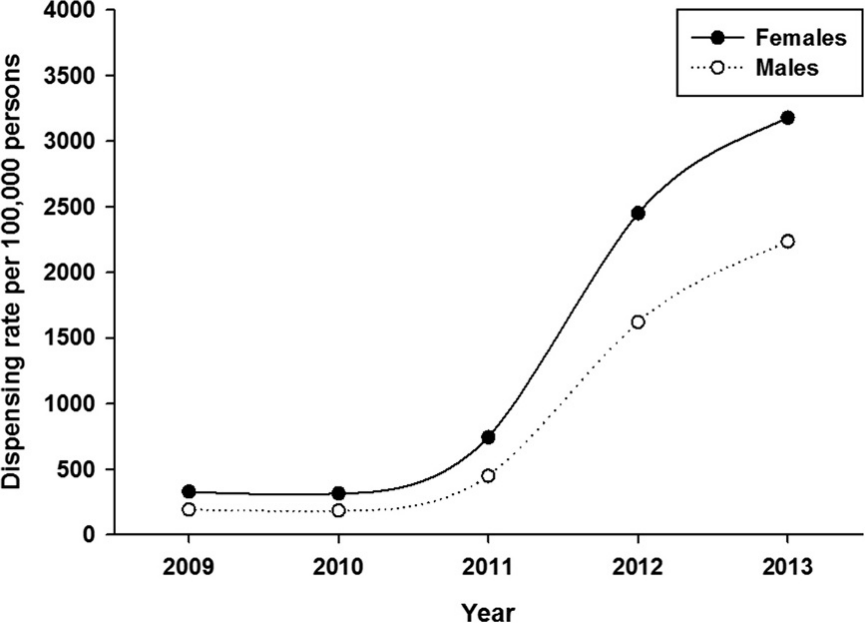
**HZV dispensing rates among persons aged 50 years or older by year by sex, Alberta 2009–2013.**

**Figure 3:**
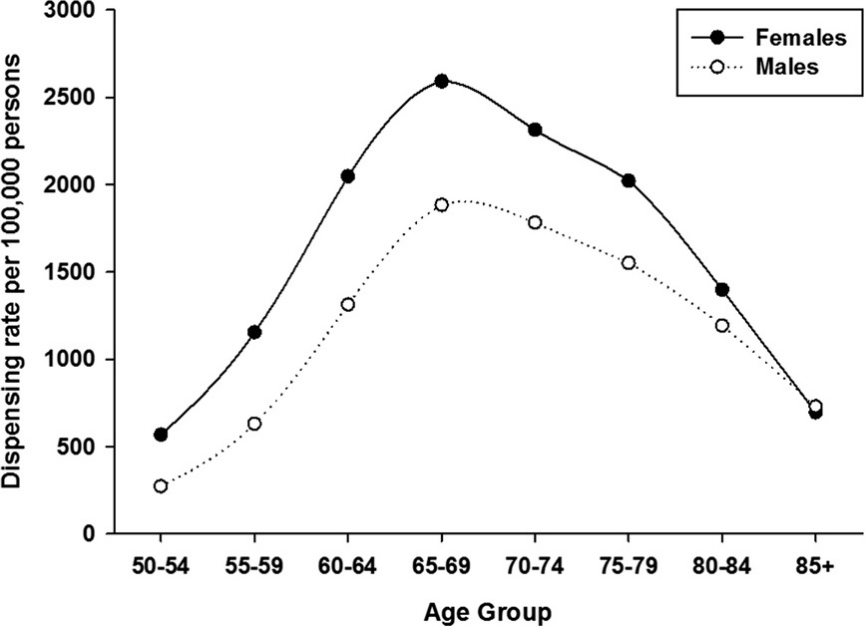
**HZV dispensing rates for females and males by age groups in Alberta, 2009–2013.**

**Figure 4:**
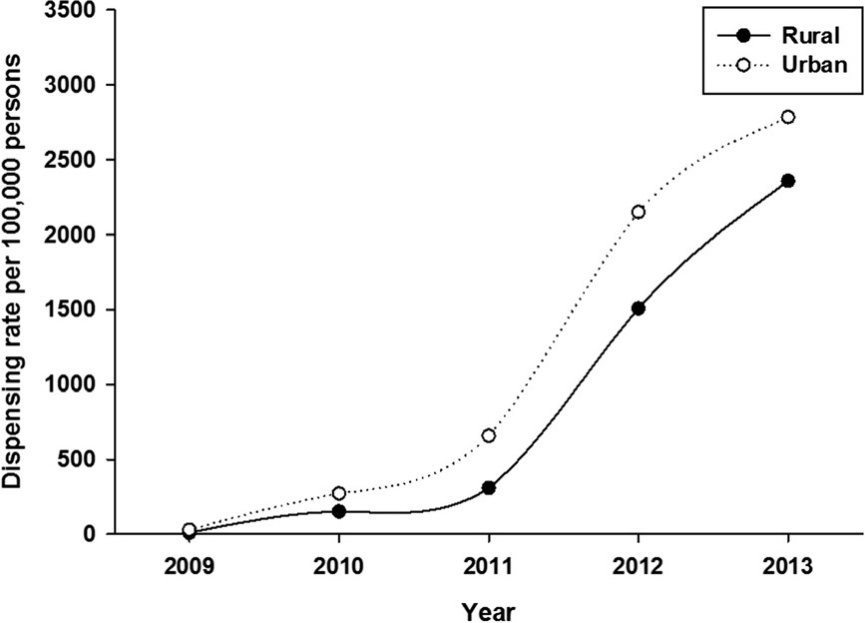
**HZV dispensing rate by geography (rural and urban communities) in Alberta, 2009–2013.**

**Figure 5:**
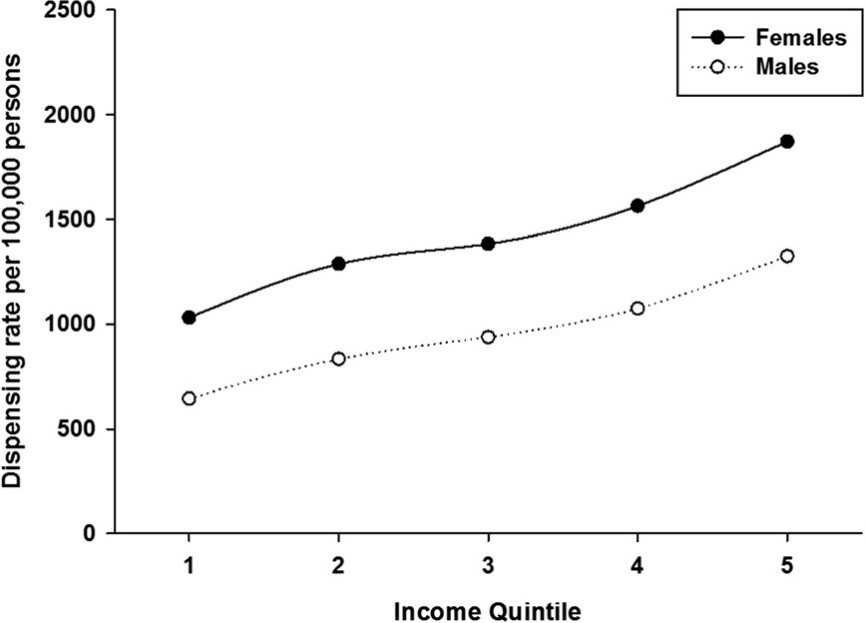
**HZV vaccine dispensing rate for females and males by income quintile in Alberta, 2009–2013.**

Table 1 shows dispensing data by age group and geography. Over the period 2009 – 2013, 69,020 unique individuals filled a prescription for HZV. This increased from 279 individuals in 2009 to 34,427 in 2013. Most of the vaccine (87%) was dispensed to urban residents. The largest proportion of vaccine was dispensed to persons aged 50 years or older. Vaccine was most frequently dispensed to persons aged 60–69 years; 42.5% of all HZV that was dispensed. About 2% of vaccine was dispensed to persons aged 5–49 years.HZV dispensing rates among persons aged 50 years or older increased every year in all age-groups (Figure 1). Within each year, the dispensing rate increased steeply among younger persons, peaked for age-group 65–69 years and then declined. Dispensing rates for both males and females began to sharply increase in 2011 but were higher in every year for females compared to males (Figure 2). However for both sexes, the pattern of increasing rates for both males and females by age group over the period was consistent: increasing to a peak for age-group 65–69 years, and then declining (Figure 3). There was a sharp increase in dispensing rates among both rural and urban residents in 2011 although rates were consistently higher for urban compared to rural residents in every year (Figure 4). As can be seen in Figure 5, dispensing rates increase for both males and females by income quintile.

**Table 1:** **Number and proportion of doses of herpes zoster vaccine dispensed by age-group and geography (rural/urban) 2009 - 2013**

Age group	N doses dispensed		Total N doses dispensed (%)
	Rural	Urban	
< 5 years – 49 years	191	1,216	1,407 (2.0)
50-59 years	1,877	14,577	16,454 (23.9)
60-69 years	3,940	25,381	29,321 (42.5)
70-79 years	2,293	13,936	16,229 (23.6)
80 years or older	672	4,842	5,514 (8.0)
TOTAL	8,973	59,952	68,925 (100)

(Geographic data were missing for 95 persons).

Taking all people that filled a prescription between 2009 and 2013 and who were still residents of Alberta in 2013, a population coverage estimate was calculated to be 8.4% for individuals aged 60 years and older. Among those aged 60 years or older, coverage was 7.2% and 9.5% for males and females, respectively. Individuals living in urban centers had higher coverage than those in rural areas (8.8% vs. 6.6%). Among those aged 50 years or older, coverage was 6.1% for the population as a whole (4.8% for males, 7.3% for females). Individuals living in urban centers had higher coverage than those in rural areas (6.2% vs. 5.3%).

The variables included in the final multivariable model were geography, age-group, sex, year, income quintile, pharmacy/population ratio, and the two-way interactions between geography*year, geography*income quintile, and age-group*sex (Table 2).

**Table 2:** Predictors of HZV dispensing rates for those aged 50 years or older*

Variables	Reference group	OR ^1^	95% CI ^2^
Rural geography	Urban	0.68	0.53-0.87
Female sex	Male	1.44	1.35-1.53
Age group	50-54		
55-59 years of age		2.14	1.96-2.33
60-64		4.71	4.33-5.13
65-69		6.64	6.10-7.24
70-74		6.50	5.95-7.10
75-79		5.86	5.35-6.40
80-84		4.13	3.76-4.54
85+		2.13	1.93-2.36
Year	2009		
2010		11.69	9.08-15.07
2011		25.97	20.24-33.32
2012		105.41	82.41-134.83
2013		153.42	119.98-196.19
Income quintile	Q1^3^		
Q2		1.18	1.10-1.27
Q3		1.34	1.25-1.44
Q4		1.48	1.38-1.58
Q5		1.57	1.45-1.69
Two-way interaction terms			
Geography*year	Urban*2009		
Rural*2010		1.31	1.02-1.69
Rural*2011		1.20	0.94-1.54
Rural*2012		1.44	1.13-1.84
Rural*2013		1.57	1.22-2.00
Geography*income quintile	Urban*Q1		
Rural*Q2		0.87	0.81-0.93
Rural*Q3		0.93	0.86-0.99
Rural*Q4		0.87	0.81-0.93
Rural*Q5		0.70	0.65-0.76
Sex*age group	Male*50-54		
Female*55-59		0.96	0.88-1.04
Female*60-64		0.90	0.82-0.97
Female*65-69		0.87	0.80-0.94
Female*70-74		0.83	0.76-0.90
Female*75-79		0.81	0.75-0.89
Female*80-84		0.78	0.710.85
Female*85+		0.70	0.63-0.77

*Adjusted estimates: adjusted for rural/urban geography, sex, 5-year age group, year, income quintile and 2-way interaction terms, ratio of pharmacies/total population.

^1^OR = odds ratio.

^2^95% CI ^=^ 95% confidence interval.

^3^Q1 = income quintile 1 (lowest income quintile).

## Discussion

The dispensing of HZV has substantially increased in Alberta over the period 2009 – 2013. In Canada, although the vaccine was licensed in 2008 and NACI indicated that the vaccine could be used in persons aged 50 years or older in 2010 [10], the vaccine was not authorized for use among people aged 50 years or older until 2011 [11]. In Canada physicians are advised, from a medico-legal perspective, that they should make patients aware of information including “official recommendations from authoritative groups, such as governments and medical specialty associations” [24]. This may explain in part why dispensing rates increased sharply starting in 2011. The increasing rates since that time may be reflective of increased patient awareness or requests for vaccine due to the influence of one or more of physicians providing information or recommendations, pharmacists providing information or vaccination services and/or direct to consumer marketing by manufacturers [14].

Rates of vaccine dispensing were lower for rural compared to urban residents, even after adjusting for sex, age-group, income quintile and ‘access to pharmacies’ (pharmacy/population ratio) in our model. However, pharmacy/population ratio is a blunt measure of access, and does not take into account that there might be a requirement to travel a greater distance to access a service in a rural compared to an urban area [25]. Rural Alberta residents have access to fewer healthcare providers per thousand population than do urban residents [25] thus it is also possible that the observed rural/urban difference in dispensing is quite simply due to a relative lack of access to physicians as vaccine must be prescribed before it can be dispensed.

In Alberta, HZV is not publicly funded and not covered by the publicly funded provincial drug insurance plan for seniors [26]. Despite the lack of public funding, we estimate population coverage among persons aged 60 years or older to be 8.4%. In 2011, among members of Kaiser Permanente Southern California (a population insured for HZV), HZV coverage among those aged 60 years or older who did not have a contraindication to vaccination was an estimated 20.7% , compared to an estimated 15.8% in the U.S. general population, a mixture of persons with and without insurance [27]. It is not surprising given the lack of public funding that Alberta coverage rates were lower than those observed in the United States; however, funding may be only one factor that contributes to the differences. Aside from the effect of other determinants of health, the promotion of vaccination can be facilitated by having population goals. In the United States, there is a Healthy People 2020 target of 30% population coverage among persons aged 60 years or older [27]. In Canada there are no national goals or targets for HZV [28].

In Canada, cost-related non adherence to prescription drug was reported by 4.8% of people aged 65 years or older [29]. The results of our model showed that persons of higher, compared to lower income were more likely to be dispensed HZV. Public funding of the vaccine might reduce the impact of income quintile on being vaccinated. In Alberta there has not been evidence of health disparity in terms of shingles by income [8]; however this was prior to the licensure of HZV. The observed association between income quintile and vaccine dispensing suggests that ongoing monitoring of shingles and vaccine dispensing by some measure of income is important as this picture may change over time in the absence of public funding for HZV.

Females were still more likely than males to be dispensed the vaccine even after adjustment for income quintile. Others have found that females were more likely than males to accept HZV [27, 30]. These studies were conducted among populations for which HZV was either covered by insurance for most participants, or provided for free. In a managed care organization in the United States where most of the study participants had insurance coverage for HZV, vaccine uptake was found to be highest in the age group 65–74 years, and higher among females than males [27], a similar pattern to what we observed. As part of an annual influenza vaccination campaign, Opstelten and colleagues in the Netherlands [30] offered free HZV in addition to influenza vaccine to patients aged 65+ in primary care practices. Females were more likely than males to accept HZV by itself or with influenza vaccine. Gender associations may reflect multiple factors including differences in perception of risk or health service utilization. In the Netherlands, reasons for refusing HZV included perceptions that the risk of shingles and/or the severity of shingles was low, that the general practitioner (GP) had not recommended the vaccine and that it was difficult to attend the GPs office for vaccination [30]. Lee and colleagues [31] studied patients attending academic primary care practices in the USA. The cost of HZV was covered by health insurance for the majority of participants. Patients who had seen a person with shingles were more likely to be vaccinated, or if not vaccinated, more likely to be interested in being vaccinated than those who had not. In the same study, participants listed reasons for being vaccinated as including both recommendations from doctor (42%) and recommendations from media/advertising (32%). Thus it is possible that Alberta females compared to males may differ in their perceptions of disease risk or severity, and/or be more likely to have received a positive recommendation to be immunized from a trusted health care provider. The incidence of shingles has been observed to be higher in females than males in studies from several countries [5, 32–35]; although it is possible that this pattern might be due to differences in health care seeking behavior. Even if the difference in disease epidemiology is an artifact of differences in health care seeking behavior, it is possible that females compared to males may differ in one or more of perceptions of risk of disease, disease severity, or having seen a person with shingles, factors found by others to be associated with accepting HZV [30, 31].

It is interesting that rates of HZV dispensing declined for both males and females aged over 70 years compared to rates for younger persons. A similar pattern has been observed by others: rates increase with age up to a point and then decline [27]. We do not think this can be explained by a decrease in physician utilization by age (a possible marker for age-related decreasing opportunities to be prescribed HZV), as physician utilization does not decrease with age among older persons in Alberta (Alberta Health, unpublished data). We hypothesize that older persons might be cumulatively more likely to have had shingles than younger persons, thus be less likely to receive a recommendation or prescription for HZV. In Canada, NACI made an explicit statement in 2010 that there was no recommendation for immunizing persons with a history of shingles [10]. It is possible that physicians interpreted this to mean that they should not recommend the vaccine to these persons. NACI changed position on this in 2014 and now states that “Herpes zoster vaccine may be administered to individuals ≥50 years old with a prior history of herpes zoster” [36]; thus the age-related pattern of HZV dispensing may change in the future.

Persons who received HZV outside of the province of Alberta would not be captured in the PIN database. However, only 1.2% of seniors moved from one province to another between 1996 and 2001 [37], thus although our coverage estimates might be underestimates, we perceive that this bias is likely to be small, particularly for those aged over 60 years.

We have assumed that doses of vaccine that are dispensed are actually administered, if this is not the case then we have overestimated HZV utilization and vaccine coverage. The PIN database captures data from community pharmacies, and thus would not capture any doses of vaccine that were dispensed from either by a hospital pharmacy or via a physician office. We think the former unlikely, but have no data to assess this. Physician offices, however, could directly order vaccine for patients and administer it at a charge to the patient. We think this unlikely to have occurred due to the complexity of storage and handling requirements for the vaccine. Zostavax™ is the sole HZV that is authorized for use in Canada, and it must be kept frozen, then used within 30 minutes of reconstitution [10]. Further, in Alberta physicians have a limited role in immunization as routine immunizations except for influenza immunization are provided exclusively through public health clinics. Thus, Alberta physicians may be less likely than those in other provinces to invest in appropriate storage facilities and staff training for a vaccine that requires more than refrigeration.

We note that HZV was dispensed to persons aged < 50 years in our data, although this accounted for only about 2% of the vaccine dispensed. This might represent off label prescribing/dispensing: use beyond the criteria covered in the approval granted by Health Canada [38]. Future research should determine if such is the case and if such practices are beneficial to the patient.

It is possible that the PIN data misclassified persons, especially those under the age of 50 years as having received HZV when a different vaccine was actually dispensed, although we think this unlikely. We perceive that the most probable error that might occur would be the entry of an incorrect Drug Identification Number (DIN), particularly that of entering a DIN for HZV when a DIN for varicella vaccine should have been entered. Varicella vaccine is publicly funded in Alberta, including for adults. Publicly funded vaccines are not dispensed through community pharmacies, thus are not recorded in PIN. Between 2009 and 2013, there were less than 100 prescriptions filled for varicella vaccine according to PIN (Alberta Health, unpublished data). Assuming this was the most common mis-coding of vaccine, the impact on overall coverage by year would be minimal. There is also a small possibility that pharmacists entered an incorrect personal health number into PIN, although this would increase the likelihood that an incorrect name (corresponding to the incorrect personal health number) would also be listed on the prescription as filled, so that such errors would be promptly identified at the time of the dispense event. This would, if not corrected at the time of the dispense by the pharmacist (pharmacists are trained to double check the names of the persons to whom dispenses are made against the prescription scrips), still result in the correct number of dispenses for the vaccine, but might introduce errors for age-specific rates. We think the impact of this, did it occur, would be small.

PIN is unique in capturing drug and biological dispensing regardless of insurance status as these data are not currently captured in the Alberta immunization registry. PIN data have proven useful in validating dispensing data for pharmaceutical dispensing from other sources [39]. PIN data, because they are not subject to recall, could also be used to validate self-reports of HZV immunization from a population survey and can contribute to estimations of population coverage with HZV.

## Conclusions

The rate of dispensing of HZV in Alberta has been rapidly increasing without public funding for this vaccine. Monitoring HZV uptake should continue regardless of public funding as it is an important component of surveillance of the impact of varicella (and of HZV) vaccination on the incidence of shingles.

## Abbreviations

GP: General practitioner
HZV: Herpes zoster vaccine
NACI: National Advisory Committee on Immunization
PIN: Pharmaceutical Information Network
PHN: Personal health number, a unique personal identifier
DIN: Drug Identification Number
OR: Odds ratio
95% CI: 95% confidence interval.
